# Digital farming as a cultural shift. Enrolling the “good farmer” through discourses on digitalization and sustainability in agriculture

**DOI:** 10.3389/fsoc.2026.1801668

**Published:** 2026-08-24

**Authors:** Marco Serino

**Affiliations:** Department of Political Sciences, University of Naples Federico II, Naples, Italy

**Keywords:** digital agriculture, discourse, ecological transition, enrolment, good farmer, precision agriculture, smart farming, sociology of translation

## Abstract

This study critically examines how digitalization in agriculture is framed in public discourse surrounding the ecological transition, considering both the information and communication of European institutions, and the discourses of the actors concerned with the promotion of innovation in this sector at a local, Southern Italian level. Theoretically, the study draws on a conceptual framework encompassing rural studies and Science and Technology Studies (STS). It relies on Michel Callon's and Bruno Latour's sociology of translation and the notion of the “good farmer” developed by Rob Burton, attempting to integrate these approaches in order to understand how the route to digitalization is discursively constructed in cultural rather than technical terms. This work is based empirically on qualitative data from participant observations in agricultural events and documents relevant to the EU's online communication about digitalization in agriculture. The findings illustrate that, in the discourses analysed, specific strategies are pursued that attempt to problematise the path of agriculture as inevitably tending to sustainability, with digitalization being presented as “interesting” for farmers as far as it enables them to maintain productivity. Indeed, as productivity is intertwined with sustainability and resilience, farmers can be considered as “good farmers” to the extent that they become able to embrace digitalization and comply with EU regulations about sustainable agriculture.

## 1 Introduction

In recent years, European agricultural policies have placed greater emphasis on digital innovation. In this context, institutional communication has developed narratives that frame digitalization as a key driver of sustainable development strategies and as part of a broader “greening” of agriculture (Wolf and Wood, 1997; Oui, 2025). However, there are critical dimensions in these narratives that deserve greater scrutiny, as they foreground the role of digital tools in addressing both current and future environmental challenges and development goals, thereby fostering pervasive processes of datafication and platformization within the agricultural sector (Van Dijck et al., 2018; Freidberg, 2020; Fraser, 2022; Sauvagerd et al., 2024).

In the extant critical literature on precision farming, it is argued that the application of digital technologies does not actually help to address environmental issues by impinging on production processes. Instead, a growing number of studies point out that such technologies serve to legitimise conventional, industrial models in this sector (Wolf and Wood, 1997; Miles, 2019; Duncan et al., 2021), while others cast doubt on the idea that big data effectively lead to improvements in sustainable agriculture (e.g. Lioutas and Charatsari, 2020).

Hence, the present study critically examines how digitalization in agriculture is articulated within public discourse concerning the ecological transition. The term “public discourse” is employed here to denote all written and oral utterances intended to engage with individuals who are affected by the transformation of a practice necessitating not only a technical but, above all, a cultural shift.

The study employs a thematic analysis of institutional documents and ethnographic material from participant observation in a farm and in public events dedicated to agriculture. It focuses on the narratives expressed at the European and regional (Southern Italian) levels by the relevant actors (advisors, researchers, trade associations, and public authorities) involved in the development of discourses aimed at legitimising digital agriculture.

The theoretical framework combines critical perspectives on precision agriculture and smart farming (Wolf and Wood, 1997; Tsouvalis et al., 2000; Clapp and Ruder, 2020; Fraser, 2022) with insights from the “good farmer” approach (Burton, 2004; Sutherland and Burton, 2011; Sutherland and Darnhofer, 2012; Burton et al., 2020).^1^ This perspective sheds light on how, in the relevant discourse, implementing digitalization in agriculture can be associated to improvements in farmers' visions and practices—also in line with the views of policymakers.

Furthermore, works in the field of Science and Technology Studies (STS) that address agriculture (Miles, 2019; Freidberg, 2020; Duncan et al., 2021), and seminal works in “classical” STS, notably the *sociology of translation* by Michel Callon (1984) and Bruno Latour (1987), provide a substantial theoretical and also methodological framework. The concepts of *translation* and *enrolment* are of particular pertinence when analysing how discourses on digital innovation in farming are performed. The concept of enrolment is employed here to denote the strategy by which different heterogeneous actors are involved in discourse with a view to underpinning specific narratives, establishing connections between them and several “allies” (Picardi et al., 2024).

The empirical study involved different settings and data collection strategies, culminating in the analysis of documents and fieldnotes. The initial phase (years 2023–2024) of the research design consisted of an ethnographic study that revolved around the role of technoscientific knowledge in agriculture. Multi-sited ethnography (Marcus, 1995; Hannerz, 2003) was thus employed to conduct several participant observations in a farm and in agricultural events. The initial findings from this work provided the foundation for the subsequent phase of the study (2025–early 2026), which focused on the collection of web-based documents pertinent to the communication of European institutions, Italian trade associations, and regional authorities regarding the directions of agricultural policies towards digital innovation and environmental sustainability.

The thematic analysis of these materials was aimed at addressing the following research questions:

–RQ1: What forms of farmers' enrolment can be identified in the discursive practices concerning digital innovation in agriculture?

–RQ2: How are the different human and non-human actors enrolled in public discourse, and in what ways they position themselves or are positioned within this discourse?

–RQ3: What kinds of associations can be identified among ideas and concerns related to digital and ecological transition in agriculture in the relevant discourses?

–RQ4: How do the discourses of different actors involved in agricultural innovation construct the figure of the “good farmer” in relation to digital and ecological transitions?

In the following section, definitions and theoretical elaborations on digital agriculture are reviewed. The ongoing debate on the application of digital tools to agriculture provides the conceptual framework to understand this transformation of farming as a matter of critical analysis, while the sociology of translation offers a valid analytical lens to investigate how this transformation is framed in discourse (namely, how the relevant actors and processes are discursively constructed to legitimise a given sociotechnical arrangement of people, things, organisations, procedures, etc.). Subsequently, the methodology and results of the qualitative study are presented, followed by a discussion of the relevant implications.

## 2 Theoretical background

### 2.1 Precision agriculture, smart farming, and sustainability

The digitalization of agriculture is a multifaceted process that is primarily characterised by the implementation of several key technologies and technology arrangements that aid farmers in enhancing the rationalisation of production (Wolf and Wood, 1997; Miles, 2019; Fraser, 2022). In this sense, the primary aim of the introduction of digital technologies in agricultural practices is not merely an improvement in automation but rather the increasing application of agricultural decision support systems (AgriDSS, or simply DSS) (Eastwood et al., 2012; Lindblom et al., 2017; Lundström and Lindblom, 2018).

Two main approaches have been identified in digital farming: precision agriculture and smart farming. Precision agriculture (PA) is closely associated with the notion of site-specific management (SSM), namely “the idea of doing the right thing, at the right place, at the right time” (Bongiovanni and Lowenberg-Deboer, 2004, p. 361; cf. Wolf and Wood, 1997, p. 180; Tsouvalis et al., 2000, p. 910). In this respect, Bongiovanni and Lowenberg-Deboer (2004, pp. 361–362) define PA as follows:

Precision farming provides a way to automate SSM using information technology, thereby making SSM practical in commercial agriculture. PA includes all those agricultural production practices that use information technology either to tailor input use to achieve desired outcomes, or to monitor those outcomes (e.g. variable rate application (VRA), yield monitors, and remote sensing).

Nonetheless, the idea of SSM is “as old as agriculture,” although it gained momentum with the 20th-century shift towards greater uniformity in intensive and large-scale agriculture (Bongiovanni and Lowenberg-Deboer, 2004, pp. 361–362). PA is also often presented as “novel,” despite the fact that its development has been ongoing since the 1990s in Europe (Tsouvalis et al., 2000; Duncan et al., 2021). Its application, however, has further expanded with the advent of smart farming.

While PA is regarded as a set of tools that can take in-field variability into account (Wolfert et al., 2017) and, consequently, cope with unpredictability, smart farming is rather seen as a management approach (Duncan et al., 2021, p. 1187). Indeed, the concept of smart farming is predicated on the interconnection of actors, institutions and practices facilitated by technologies related to big data, the Internet of Things (IoT), cloud computing, robotics and artificial intelligence (Wolfert et al., 2017; Klerkx et al., 2019). In the words of Clapp and Ruder (2020, p. 49):

Digital farming (also known as “smart” or “digital” agriculture) connects farm equipment to software platforms that track on-farm data and enable analyses of soil and climate conditions in specific location in order to provide farmers with advice regarding seed choice and more precise application of pesticides and fertilisers.

Furthermore, Clapp and Ruder contemplate the integration of genome editing within the paradigm of digital farming, involving the use of “big data generated from computer-assisted genomic mapping to determine edits to the DNA of living organisms that promise a more precise way to modify a plant's genetic code to express new traits that can improve crop performance” (Clapp and Ruder, 2020, pp. 49–50).

A fundamental issue in the discourse concerning digital agriculture pertains to the question of whether it offers tangible benefits to farmers or the environment, and the manner in which its implementation is associated with sustainability. Digital agriculture is predicated on two fundamental promises. Firstly, it assists farmers in their day-to-day work, thereby mitigating the uncertainties that are inherent in their profession. Secondly, it is characterised by the “technical precision” that is facilitated by digital tools, which consists in promoting “more efficient and environmentally friendly farming practices” (Oui, 2025, p. 2729). Analogously, digital farming and gene editing are regarded by their proponents as being interlinked in an “environmental promise” (Clapp and Ruder, 2020, p. 55).

Nonetheless, the relationship between digital agriculture and sustainability is quite controversial. The point raised by social scientists about PA revolves around the legitimation of productivist regimes in agriculture through digital tools, “vis-a-vis environmental concerns” (Duncan et al., 2021, p. 1183). As Wolf and Wood (1997, p. 184) noted, social movements centred on environmental concerns contrasted the development of an industrial agriculture, and this conducted to the “greening” of agriculture, the same greening that informed the Common Agricultural Policy (CAP), that is, the “environmental turn” that led public policies to transform agriculture “in order to reconcile environmental sustainability with farm productivism” (Oui, 2025, p. 2727). The promised precision of digital farming technologies and big data is also subject to criticism, as these technologies might in practice be affected by inaccuracies. In fact, expectations of promised precision might contribute to a “precision trap” (Visser et al., 2021).

In their critique of precision farming, Wolf and Wood (1997, p. 185) observed that, in the context of mounting social pressures associated with environmentalism, there was “a rhetorical effort to portray agribusiness as significantly engaged in the environmental reorientation of agriculture.” Hence, they found that “precision farming has less to do with mitigating agricultural pollution than it does with advancing industrial modes of production.” Instead of avoiding or reducing chemical use in farming, the advocates of PA would tend to legitimise it by means of the rhetoric of efficiency, a notion that is currently associated with the “algorithmic rationality” of PA (Miles, 2019). This approach aligns with the “sustainable intensification” in agriculture, “which has the overreaching goal to increase food production from existing farmland while minimising the pressure on the environment” (Lindblom et al., 2017, p. 310; Garnett et al., 2013).

Central to PA are, then, the dimensions of rationalisation and efficiency that characterise conventional production systems (Wolf and Wood, 1997; Miles, 2019). In the empirical analyses presented in this article, these dimensions are explored through the discourses of the promoters of digital technologies for improving sustainability, who appear to support a “sustainable intensification” of agriculture as a way to cope with the challenges represented by the environmental crisis without neglecting productivity (Miles, 2019; Duncan et al., 2021).

It is important to note that a competing strategy that can be beneficial in preserving environmental resources and thus improving sustainability in agriculture is *agroecology*. This term refers to practices that “valorise in the best way ecological processes and ecosystem services in integrating them as fundamental elements in the development of the practices, and not simply relying on ordinary techniques, such as chemical fertiliser and synthetic pesticide application or technological solutions, such as genetically modified organisms” (Wezel et al., 2014, p. 3). However, a central tension exists in the fact that both agroecological approaches and digital agriculture present environmental preservation as a key objective, despite the overtly divergent positions of the advocates of these approaches (Clapp and Ruder, 2020, p. 50). This has to do with the interplay between “two transitions in agriculture, ecologisation and digitalisation” (Bellon-Maurel et al., 2022, p. 2) and its connection to sustainability, which characterises discourses of innovation in agriculture. Nevertheless, these discourses are far from homogenous and present several tensions, as is the case with PA and big data in farming (Fleming et al., 2018; Duncan et al., 2021), the opposition between digital agriculture and agroecology (Clapp and Ruder, 2020), and perceptions of the interplay between digitalization and ecologization in organic vs. conventional farming (Schnebelin et al., 2021).

### 2.2 Digitalization and the “good farmer” perspective

The present argument can also be explored by considering the cultural dimension of farming and the tensions that, in this respect, may derive from the digital transformation of agriculture. Indeed, digitalization can be regarded as a means to realize the idea of the “good farmer” (Burton, 2004), a concept that has been fruitfully applied in rural studies (Burton et al., 2020). Being a “good farmer” means, for those working in agriculture, positively evaluating one's own competence and skills. The concept is understood as a “commonsense category used by farmers to refer to a farmer whose farming behaviours reach a certain level of competence” or as an “emic category used by farmers to describe the social significance of other farmers' work” (Burton et al., 2020, p. 7).

A basic tenet of this theoretical perspective is that the “physical appearance of the crop” may hold significance for farmers, as it facilitates the assessment of their peers' work through visual perception. This feature of agricultural landscape thus functions as an indicator of good farming. For instance, an “irregular crop density or ‘crop lines' may suggest problems with the drilling of the crop” (Burton, 2004, p. 201). Through the assessment of crop characteristics such as colour and height, farmers can associate these features with some specific aspects of farming. Indeed, in doing this, they make exactly what precision farming does, albeit in a radically different way and relying on their experiential knowledge.

To a certain extent, the practice of farmers monitoring each other's performance is being supplanted by digital technologies (Wolf and Wood, 1997). The physical characteristics of crops and farmers' visual perception can be likened, respectively, to the parameters that need to be monitored and measured and the digital tools through which these data are collected (e.g., satellites, drones, or sensors) and analyzed. As far as the ability of farmers to judge one another's husbandry is based on the human perception of the conditions of landscapes and crops, this may lead to substituting socially mediated evaluation with the data collection and visualisation technologies offered by digital agriculture. This is linked to the underlying process that Wolf and Wood (1997) referred to as “the commodification of information” in agriculture, which implies the “substitution of capital for farmers' experiential knowledge” (Wolf and Wood, 1997, p. 203). This is an important point in discussions on digital agriculture, as this substitution can impinge on the definition of the “good farmer” by the farmers themselves, as well expressed by a farmer interviewed in Tsouvalis et al.'s (2000, p. 916) study: “If you have to rely on this technology *you're not a good farmer*, if it gets hard, you won't survive” (emphasis added).

Another aspect that farmers value as an indicator of farming skills is crop yield, which turns out to be “a symbol of farming ability” (Burton, 2004, p. 202). Indeed, the advantage of considering this outcome of agricultural practices is that “it provides a standardised measure of nurturing ability that can be judged on a year by year basis and between farms within the same region.” As such, Burton notes, crop yield can function as a reliable indicator of “good farming”; relatedly, increasing production provides “the core of any definition of a ‘good farmer”' (Burton, 2004, p. 202). In fact, productivity was “a post-war civic duty” (Burton et al., 2020, p. 3) that characterised agricultural policies prior to the shift occurred in the 1990s, when the reforms to the CAP led to reducing the subsidies associated with productivity and encouraged “more environmentally beneficial farming practices” (Ward et al., 2008, pp. 118–119). This is a pivotal topic in addressing the discourses of the promoters of digital agriculture, as they tend to foster the “ambition of the CAP to reconcile production and the environment” and, consequently, to orient the focus of the CAP on “sustainable intensification” (Oui, 2025, p. 2731).

### 2.3 Translation, problematization, *interessement*, enrolment

The positioning of farmers, technologies and other relevant actors in discourse can be investigated by leveraging some notions provided by Michel Callon and Bruno Latour, such as those of *translation* and *enrolment*. Focusing on the attempts to solve economic and scientific controversies, such as the famous case of the ‘domestication of the scallops' of St. Brieuc Bay (France), Callon (1984, p. 203) defines *translation* as a process “during which the identity of actors, the possibility of interaction and the margins of manoeuvre are negotiated and delimited.” This process, in Callon's formulation, first entails the “problematization,” that is, the strategy by which certain actors define their identities and the identities of other actors in order to render themselves indispensable in the network of alliances necessary for the achievement of specific objectives (Callon, 1984, pp. 204–205). Accordingly, “the problematization describes a system of alliances, or associations, between entities, thereby defining the identity and what they ‘want”' (Callon, 1984, p. 206). This also implies that the actors who attempt to establish such problematization seek to position themselves as “an obligatory passage point in the network of relationships they [are] building” (Callon, 1984, p. 204). An *obligatory passage point* (OPP) is thus defined as a “crucial step” that renders these actors indispensable and allow them to consolidate the associations they aim to establish (Minniti and Magaudda, 2025).

Dealing with the construction of scientific facts, Latour (1987, p. 108) also provides a definition of *translation*: “I will call translation the interpretation given by the fact-builders of their interests and that of the people they enrol”. Indeed, a related concept in this framework is that of *interessement*, which, for Callon, “is the group of actions by which an entity […] attempts to impose and stabilise the identity of the other actors it defines through its problematization” (Callon, 1984, pp. 207–208). As both Callon (1984) and Latour (1987) put it, the word “inter-esse” means exactly that something important for the actors involved is placed on their own way to obtain what they “want”: “‘interests' are what lie *in between* actors and their goals, thus creating a tension that will make actors select only what, in their own eyes, helps them reach these goals amongst many possibilities” (Latour, 1987, pp. 108–109; see Callon, 1984, p. 208). *Enrolment* is directly linked to *interessement*, as it “designates the device by which a set of interrelated roles is defined and attributed to actors who accept them. Interessement achieves enrolment if it is successful” (Callon, 1984, p. 211).

“Translating interests” thus consists in the set of strategies through which science and technology become embedded in society by enrolling people in sociotechnical changes.^2^ Indeed, the adoption of innovations stems from the mutual alignment of the interests of the relevant social actors. A famous example provided by Latour (1987) concerns farmers: Louis Pasteur needed to enrol farmers' interests to protect livestock in his endeavour towards the development of a vaccine against anthrax. At that time, this technology was new to farmers, but it would assist them in dealing with animal diseases.

This set of concepts is of paramount importance in this context, as it is through *interessement* that the human and non-human entities implicated in promoting innovations in agriculture are enrolled in discourse, thanks to the associations that are forged by those who act as representatives or spokespersons (Callon, 1984). Therefore, in this context the notion of enrolment is employed in the form of “discursive enrolment” (Picardi et al., 2024). Enrolment may imply the positive evaluation of certain agricultural practices that are deemed to be “good” and, as far as farmers are enrolled, it may be questioned whether and how such enrolment involves more or less directly the adoption of digital tools in agricultural practices. In this way, the concepts of translation, problematization and enrolment are placed in dialogue with the “good farmer” framework (Burton et al., 2020).

## 3 Materials and methods

In this study, the empirical analysis is aimed at comparing discourses performed at different levels of institutional communication, relying on several sources of qualitative data on those discourses. Specifically, this work concentrates on the discourses delivered by European institutions, national trade associations, and sector experts or researchers. Firstly, given the emphasis that policymakers place on digitalization in agriculture, discourses relevant to the EU strategies to promote digital agriculture are observed through information and communication websites. Secondly, these discourses are compared with those enacted by local representatives and experts of the agricultural sector in public events. The triangulation of sources provides a more robust empirical grounding for the argument of the study (Hammersley and Atkinson, 2019).

**Figure 1:**
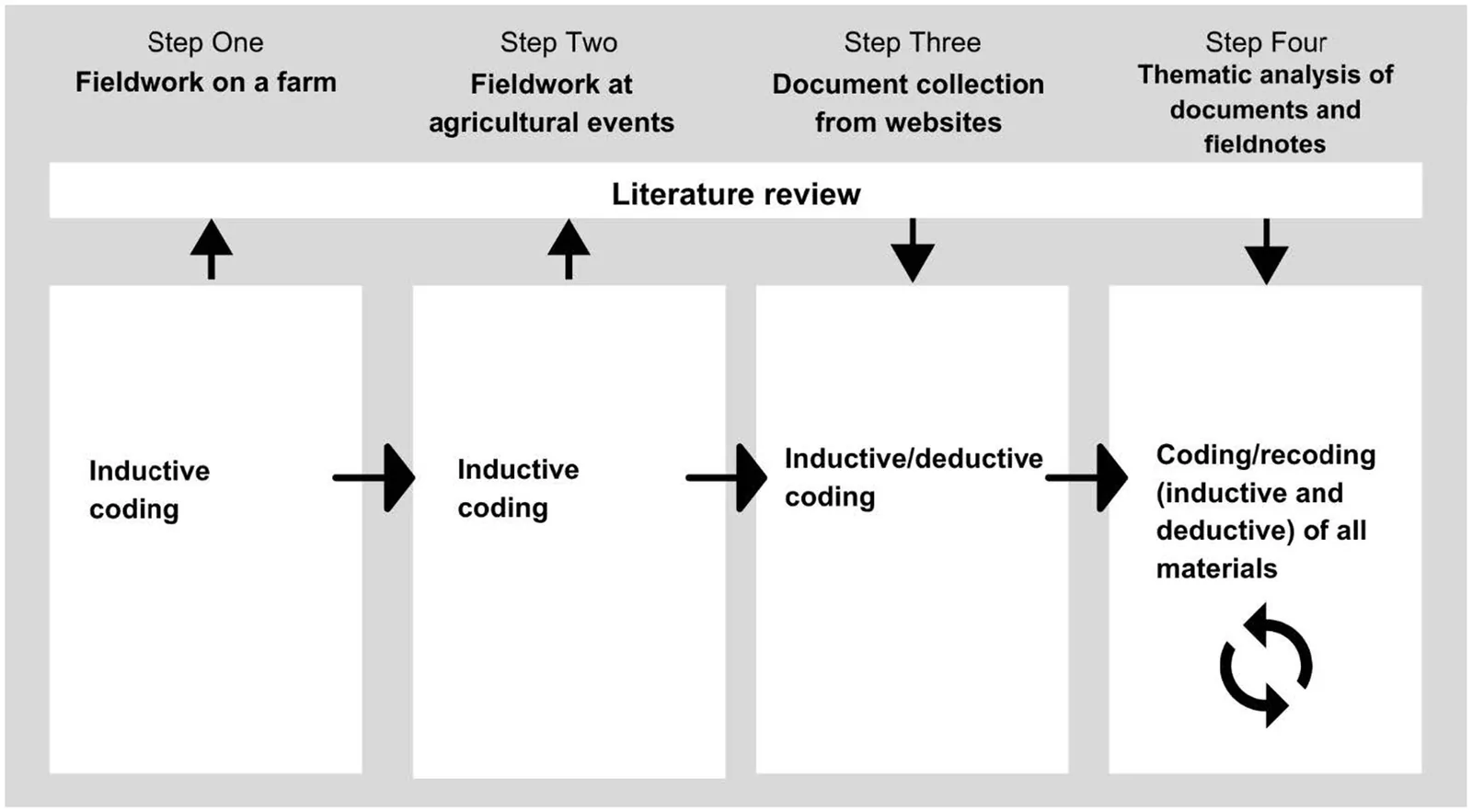
Data collection, coding process and analysis.

The methodological strategy is comprised of four steps (see Figure 1) and draws on two forms of data collection. In the first two steps, the author relied on multi-sited ethnography (Marcus, 1995; Hannerz, 2003), undertaking participant observation across diverse settings between 2023 and 2024. The empirical study was initiated with several visits to a farm (step one) located in Italy's Campania region in Spring 2023, with observation sessions that contributed to launching the research by relying on the experiential knowledge of an agricultural entrepreneur (“the Farmer,” henceforth), who acted as a key informant on the relationship between agriculture and sustainability. Moreover, a number of conversations held with the Farmer during this fieldwork have been recorded and transcribed. Indeed, the encounter with the Farmer not only allowed the author to gain first-hand knowledge of the critical facets of sustainability in agriculture, but also provided case-specific empirical evidence and was instrumental in establishing the subsequent research itinerary. This preliminary and preparatory fieldwork was also coherent with the initial question as to how technoscientific knowledge engages with farming practices.

The early ethnographic material resulting from the first research step also provided some guidelines for the literature review in both the social and agricultural sciences, which began and proceeded along with the fieldwork. In addition, the Farmer advised the author to attend a conference on the CAP 2023–2027 held during one of the agricultural events chosen as fieldwork settings, described as follows.

In the second step of the research, participant observation sessions were conducted during several public events. The present study considers materials from two of these events, which were held between 2023 and 2024: a local agricultural fair (Event 1, one-day observation), and a large and variegated agricultural exposition (Event 2, one of the largest such events in Italy, with a two-day observation).^3^ While Event 1 had no purpose other than exhibiting agrifood products, services and agricultural instruments (especially machineries), Event 2 offered the opportunity to explore how innovation is framed in discourse and voiced by representatives of trade associations, scientists, and advisors participating in the event. Indeed, Event 2 included a conference on the CAP, in addition to a conference on precision farming. Speeches delivered during Event 2 have been recorded in part, and the transcripts used to complement ethnographic data. All the recorded material has undergone a process of anonymisation.

The coding of fieldnotes was chiefly inductive, with codes being added throughout the reading of the material. Concurrently, a range of other codes emerged from a literature survey in both the social and agricultural sciences, providing a set of theory-driven codes for the subsequent coding of documents and, in the final step, of all other materials as well (see below).

The third step of the data gathering strategy involved the collection of documents (texts and images) from several websites of the relevant institutions (European Union, European Commission, the regional authority of Italy's Campania, and trade associations). The focus on the communication of these institutions is motivated by the context of the study, namely the agricultural sector in Italy, and the jurisdiction over agriculture in the territories of the European Union, as is the case with the Common Agricultural Policy (CAP).

The document collection was conducted between 2024 and early 2026, gathering more than 50 documents^4^ from the following EU websites: https://commission.europa.eu/index_it, https://agriculture.ec.europa.eu/index_it, https://digital-strategy.ec.europa.eu/it. A series of queries has been executed in the search engines of these websites, using several combinations of the keywords “agriculture,” “digital,” “digitalization,” “innovation,” and “technologies.”^5^ The search on trade associations' and the Campania region's websites was made by focusing on the pages devoted to PA and digital agriculture.

The coding of the documents was both inductive and deductive, based on the themes that emerged from reading the documents, analysing ethnographic materials, and conducting a literature survey. This implied that the coding was both data-driven and theory-driven, focusing on several general themes: the dimension of control (understood as the digital monitoring of the parameters relevant to farming activities and a correlate of efficiency), which informs almost all processes of digitalization (the latter is itself a theory-driven code); the wider theme of innovation; and the idea of productivity. A predefined code based on both ethnographic and literature materials is “data,” which is not simply understood as an obvious element of digital agriculture, but as a central actor in its own right. Key data-driven codes derived from both ethnographic materials and documents include “resilience” and “farmers” (which also covers expressions such as “*our* farmers”).

The three aforementioned steps culminated in a inductive/deductive thematic analysis (Boyatzis, 1998; Fereday and Muir-Cochrane, 2006) of all materials (step four), undertaken via a recursive coding and recoding of these materials in order to identify relations between codes across the datasets. The coding was performed using the QSR *Nvivo* software. Interrogating the empirical material by searching for coding overlap enabled the author's exploration of the interplay between different key ideas, thereby revealing how the different actors are positioned in discourse.

## 4 Results

### 4.1 Coping with incertitude, or: the calendar becomes unreliable

A common trait of the discourses investigated in this work is the attention paid to the conditions deriving from the impact of climate change on weather variability, which results in uncertainties about *when* the different mechanical or chemical treatments must be operated on plants. This is the first lesson I have “learned” from the Farmer:

He describes some strategies to cope with such incertitude, including several technical specifications of the procedures he employs in terms of environmental sustainability. For example, there are treatments that use insect traps, which must be applied at the right time and allow you to target only specific insects that are harmful to plants. In fact, one chief example is that of bees (he says this while showing me some bees lying on flowers grown in the field). If we do not treat crops *precisely*, we risk, for example, killing bees. In general, therefore, the point is to apply *targeted treatments* with specific programming: that is, to carry out these treatments at the right time (fieldwork on a farm, fieldnotes, 14 March 2023).

Notably, what is described above is nothing but the rationale of SSM, which is, in fact, an old idea (Bongiovanni and Lowenberg-Deboer, 2004). The issue is still the principle of the targeted adoption of processes and inputs aimed at treating crops, without wasting resources – or, in the words of the Farmer, “without *wasting nature*” –, without damaging unaffected areas of the land or crops, and avoiding impacts on animals as well, thanks to the *precision* of the treatment. This can be obtained in two ways, namely agroecological practices, as described in the excerpt above, and PA:

Regarding PA, the Farmer shows me a part of his land that is cultivated with wheat. He points out some strips of land where the grass is yellowing. Then, he underlines that, as there could probably be some problems in that area of the land, these problems could be addressed using PA tools by treating *only that layer of soil* and not the entire crop, thanks to the information provided by the drone (he describes the flight of the drone in the field), or through sensors installed in the ground (fieldwork on a farm, fieldnotes, 14 March 2023).

Precision agriculture, in the Farmer's words, is “agriculture done with very, very localised treatments”, a method that he said he did not employ at the time of fieldwork. Rather, his strategy was that of integrated pest management (IPM), a regulated practice entailing minimised use of pesticides (Stenberg, 2017). Overall, his view of farming appeared in line with agroecological principles: “I'm talking about agriculture... Must we call it natural? Must we call it of... protection of the land? Not exploiting the soil too much?” (the Farmer, audio transcript, 26 May 2023). This relates to sustainable agricultural practices involving the rationalisation of inputs without the use of digital tools.

Nevertheless, the Farmer said that he was keen to adopt PA one day. According to him, “*agriculture done right*, precision agriculture *is the future*. This is *our future*” (the Farmer, audio transcript, 22 September 2023). Reflecting on this statement in the light of the “good farmer” perspective (Burton et al., 2020) helps to understand how such a view of precision in information about the processes and functions of agricultural production, aiming to allow targeted actions on lands and crops, might be said to be “good”. The future, in the Farmer's view, is the adoption of procedures that enable one to treat crops with as few chemicals as possible – without avoiding them at all – harmonising technical means with the life cycle of plants, and trying to cope with the challenges imposed by climate change, first of all in terms of the timing of chemical treatments. This entails abandoning the so-called farming “by calendar”, “which means that we do treatments when they are not needed” (the Farmer, audio transcript, 26 May 2023). Being attentive in this way to variations in timing and coping with uncertainty can thus be likened to “good farming”.

Obviously, the case of this farmer is anything but representative of the wider population of Italian or European farmers. Conversely, the Farmer's perspective appears to align in part with the narrative articulated by the experts and representatives of trade associations who participated as speakers in a conference on the CAP held during Event 2.^6^ Those speakers attempted to *translate* the push for innovation through digital technologies to make it *interesting* for farmers.

In that conference, measurability appeared one the most important ideas employed to enrol farmers' interests. A representative of a trade association began their talk by referring to a picture showing a farmer seen from behind, facing the sun in a field, with a text alongside reading “We can improve only what we can measure”. This suggests that the production and management of information are deemed pivotal in defining the present and future of farming. Relatedly, the representative wanted to highlight that the path to innovation must be informed by the capability of controlling all the key variables relevant to agricultural practices. Hence, they referred this capability, metaphorically, as to the dashboard of a car: analogue to the latter are the tools that farmers need to master in order to keep those variables under control, a “console” that enables the measurement of changes in parameters such as temperature, humidity, etc. (i.e. the in-field variability). The reason behind this, they went, is that the parameters that were once “normal” and more manageable are now undergoing variations that no longer allow treatments to be carried out exclusively “by calendar” – that is, according to seasonal weather conditions as expected. Therefore, they added, today farmers need to have “the courage to start using small-scale technologies” that enable them to control this increasing uncertainty (Event 2, fieldnotes).

### 4.2 Good farming is not “traditional” farming

Across the different ethnographic sources, what can be termed “traditional” agriculture appears to have specific meaning. The Farmer argued that by “traditional” agriculture, we must not intend agriculture made without any technical means, and therefore without physical-chemical treatments. On the contrary, “traditional” agriculture is one which is done using chemical treatments, irrespective of sustainability concerns and the increasing in-field variability due to climate change. Therefore, it is far from being “agriculture done right”, as noted above. It is also very different in meaning from “traditional” agriculture intended as an opposite to industrialised modes of production. In this sense, speaking of traditional agriculture would even contrast with the “environmentalization” of agriculture (Buttel, 1992; Wolf and Wood, 1997).

To interpret this shift in meaning—which is, indeed, a form of translation—it is worth recalling the question of the unreliability of the calendar. Agriculture “by calendar,” in the Farmer's opinion, is synonymous with traditional, or conventional, agriculture: “This year, if we want to have olive oil […] we have to be careful, [we would need] some treatment in August, if we have a vintage like the last year. So last year's vintage makes it clear that *traditional* [he says it emphatically] *agriculture, by calendar, does not work*” (the Farmer, audio transcript, 26 May 2023). Hence, traditional agriculture is equated with an inefficient practice that avoids rational means and, consequently, relies on the calendar to schedule chemical treatment, as already illustrated above.

This trend seems to align with the one emerging from participant observation in agricultural events. Indeed, what the Farmer was talking about is a shift in farming culture, something that is shared with other views about the adoption of digital technologies in agriculture. This idea of traditional or “simple” agriculture, in contrast to technologically advanced farming, was also apparent from an interaction involving the author during Event 1, in which agricultural machineries were exposed. It is worth quoting the related fieldnotes, as the content of the conversation resonates with Tsouvalis et al.'s (2000, p. 910) description of yield metres on combines:

I ask a person providing information to passers-by about a large machine, which is used for both sowing and tilling the soil […]. I ask them if this operation is carried out “intelligently,” if the machine is equipped with GPS. They tell me that what I'm talking about is a device which provides information on the soil conditions in terms of yield. In other words, it can detect information on how much a given section of the land has produced and saves this data to a USB drive, which can then be read by a computer, thus making it possible to map the land using colours to distinguish areas based on yield (“for example, you might use red to indicate a certain yield, etc.”). In this way, using a programe, you can measure out the fertiliser or other necessary materials. However, they add that the other thing I might be referring to is auto-steering, which, however, is used on tractors and is based on satellite data. Then they tell me: “Actually, we need to go even further back,” as the issue of yield and the data collected on it using the system they describe involves the use of the combine harvester, which is employed to initially gather the aforementioned data. A person standing next to them, who had been following the conversation, adds: “Well, actually, you have to go back even further, because you need to look at this [yield calculation] over the previous 4 or 5 years; only then can you adjust accordingly.” After a while, leaving the conversation, the person who gave me all that information says to me: “*This isn't the simplest agriculture!*” (Event 1, fieldnotes).

Once again, simple or traditional farming is contrasted with high-tech, data-driven farming. The cornerstone is the quantity and quality of information available to farmers to help them do their job effectively, but this does not necessarily mean equating digital agriculture with “good farming.” As mentioned in the previous section, there is a view of sustainable agriculture based on rationalising inputs and treatments, which is not necessarily linked to precision farming. The point of view of the Farmer is illustrated by the following excerpt:

Author: “But then when you talk about traditional agriculture...” Farmer: “Traditional is the classic farmer that... I'll give you an example, you now go to the area of [name of the area of the region], ok? You don't see the grass under the plants, you see the bare and raw soil from outside. […] That is a crop that will have to disappear. Because you are taking organic matter away from the soil. So, if the day after tomorrow morning you come to say that the mountain has collapsed… do you see this? [compares to his soil] It's a clay soil, so last week you would have sunk. There you can still see that you would have sunk. Ok? But with the grass I could enter, I walked, I had no problems because the soil made it firm for me. So, if we begin to reason that grass is there, it is necessary, not under the plants, because it leads to the asphyxiation of the roots. But in the centre, I must keep it” (conversation between the author and the Farmer, audio transcript, 26 May 2023).

The above conversation illustrates how a farmer's skills enable them to assess the condition of the land and distinguish between good and bad farming practices. This experiential knowledge is a way of “seeing through the ‘good farmer's' eyes” (Burton, 2004), namely the ability to evaluate the appearance of the land. Interestingly, the Farmer associates “traditional” farming with “bad” farming, contextualising the distinction between the two in terms of a series of ecological connections: “I have less exploitation of the soil, less evaporation, more organic matter, less heating of the soil, the grass develops, the bee can come and eat the flower; that is, in the end I also safeguard the territory” (the Farmer, audio transcript, 26 May 2023). Therefore, according to the Farmer, moving away from “traditional” agriculture is the right approach. In some sense, this may be interpreted as a “good farming” perspective. Comparing the above examples from two fieldworks with a third might help to explain how this aversion is cultural rather than merely technical.

During Event 2, in a conversation with an advisor who was an expert in PA, I discussed the idea that a current trend is to move beyond the concept of “traditional” agriculture, based on the previous fieldworks. That expert agreed, and enthusiastically shared a memory of a professor who once told him that “tradition is an innovation that has won.” He wanted to mean that a cultural shift is needed to implement a digital transformation in agriculture. As a new habit, prediction should enter the realm of farming analogously to the way people look for weather forecasts. Indeed, the advisor emphasised the transformative nature of PA as a transition from an agricultural model based on experiential knowledge, which is unsuitable for the management of unforeseen events and variability, to one which is based on data-aided prediction. In addition, I shared with him my interpretation that PA techniques enhance human eyes and hands. He agreed, pointing out that this could potentially be beneficial for social inclusion (Event 2, fieldnotes; see also below).

The conference on the CAP (Event 2) featured further instances of discourse on innovation centred on a cultural shift. A representative of a trade association emphasised the significance of knowledge and scientific research in addressing the challenges posed by globalisation. Furthermore, a researcher insisted on the need to address the current changes with a view to mobilising social (that is, cultural) forces:

[This is] a transition that for simplicity we call ecological but in reality there is much more underneath because it is an ecological transition. Therefore [we deal with] the whole production technique, but it is the main transition of agriculture, it is also a social transition, because today we are asking agriculture for such a profound change that, without cultural innovation, without social innovation, this is not possible (researcher, Event 2, audio transcript).

Nevertheless, in these discourses, productivity is also highlighted, by virtue of the device of *interessement*, with utterances such as “producing food,” “producing more” or “staying on the market” (all of which were expressed during the conference) standing as key drivers in the enrolment strategies of these spokespersons. Such discourses also offer several examples of problematization: they invite farmers to change their ways of doing agriculture and suggest that implementing innovations which require them to embrace a technological turn might be useful to them for doing “good farming”, with an eye to increasing sustainability and, at the same time, to remaining productive (what the farmers “want”). The subsequent sections will seek to explore this trend in greater depth.

### 4.3 Positioning farmers in discourses on innovation: tensions and ambiguities

What are the challenges of agriculture? Producing food. And it is not easy because you must produce it with a view to a development that must be sustainable, but with an approach that must be economically viable (researcher, Event 2, audio transcript).

The above excerpt illustrates how the concepts of productivity and sustainability are intertwined in discourse, along with farmers' need to cope with production costs. In addition, the researcher quoted above underlined that “agriculture goes back to producing food.” Apparently, they meant that this is happening after a period in which that goal was not a central one: “Because for years we thought we had to focus solely on the environment, the landscape, culture and so on” (researcher, Event 2, audio transcript). They probably referred to the “shift from productivism to post-productivism” occurred during the 1990s and 2000s (Ward et al., 2008) and virtually suggested reversing in some way this trend. In fact, productivist goals are placed at the core of the discourses of these experts, fostering the *interessement* of farmers.

The importance of ensuring sustainability and productivity at the same time is expressed in EU policy discourse as well. For example, by associating data accumulation and sharing and the notions of sustainability and efficiency, EU institutions promote digital technologies and attempt to enrol farmers' interests, offering them the opportunity to compete and, concurrently, operate sustainably. The following excerpt illustrates this:

With the collection and processing of information, technological automation and robotisation, farmers can contribute towards a more sustainable agricultural production. They can *increase the efficiency and competitiveness of the farming sector, while lowering its environmental footprint*. Digital technologies can also contribute to the development of rural areas by providing better accessibility and connexions in line with the Long-Term Vision for the EU's Rural Areas (https://agriculture.ec.europa.eu-vision-agriculture-food/digitalisation_en, accessed 18/09/2025, emphasis added).

The above nexus between efficiency and competitiveness resonates with part of the speeches delivered at Event 2, when an agronomist and digital agriculture advisor said that “sustainability does not necessarily mean to produce less. […] Absolutely not, it is a myth to be debunked. Agriculture 4.0 helps us, it must help us, to produce more” (agronomist, Event 2, audio transcript). Indeed, as noted by Duncan et al. (2021, p. 1190), “increasing yield appeared to be the most common goal of precision agriculture.” Notably, this goal is one of those which, once achieved, make a farmer amenable to be deemed a “good farmer” by their peers (Burton, 2004).

Notably, the need “to stay on the market” that was stressed by a trade association representative at Event 2, during the conference aimed at promoting—though at the same time criticising (see below)—the CAP, suggests that competitiveness is displayed as something valued by farmers' spokespersons. In the latter's discourse the tone is, however, one which aims at invoking farmers' responsibility and responsiveness and, at the same time, at reassuring that their interests are not ignored. Hence, farmers' interests must change at least in part to adopt innovations while seeing their goals (i.e., to keep their business economically viable) fairly understood. To strengthen this stance, a series of translations must occur in the way farmers' identity is depicted in the discourses of representatives and experts.

Amongst the benefits of “digital and data technologies in agriculture” mentioned in one of the documents, explicit enrolment of farmers' interests is visible, and particularly one which points at the opportunity for future farmers to reduce their physical and mental workload. This is wielded to foster farmers' *interessement*: “Automation and optimization through digital technologies, including robotics, reduce the physical and mental workload of farmers, leading to improved working conditions” (https://digital-strategy.ec.europa.eu/en/policies/digitalisation-agriculture, accessed 12/09/25). Analogously, during Event 2, the leader of a trade association said that “It is *no longer the agriculture of the past*, today there are many innovations that allow us to carry out *a job that years ago was very hard, very burdensome and these new technologies can alleviate it*” (member of trade association, Event 2, audio transcript).

This kind of *interessement* appears also in the way farmers are depicted as young, confident, technologically skilled, and careful with animals in the narratives that EU's institutional communication intends to convey about the future of agriculture. A case in point is a young farmer smiling at a cow and handling a tablet—which seems to stand for a mediating tool between human agents and the object of their activity—portrayed in a European Commission webpage (https://digital-strategy.ec.europa.eu/en/policies/digitalisation-agriculture, accessed 12/09/25). Most visual representations of farmers in this communication strategy are of such tenor: digital tools populate the fields as new allies, and farmers themselves are enrolled with their interests.

Another form of *interessement* lies in the potential of mediation that PA holds as a DSS vis-à-vis the role of the farmer as someone who utilises, but is not substituted by, digital tools:

In addition to data processing, we need to start *getting support from technology, even to decide*. […] We hear more and more about DSS, Decision Support Systems. We must have *the humility* to get help from technology as well. *The final decision—we always say this—is up to the farmer, that is, the machine cannot do what the farmer does, but it can give us a big hand. It can help us interpret the data* (agronomist, Event 2, audio transcript).

In the above transcript, farmers' identity is referred to in an apparently contradictory manner. The utilisation of DSS has the capacity to function as either a potential form of displacement or an enhancement of farmers' capabilities. On the one hand, farmers must acknowledge their constrained capacity to manage information. Interpreting the data is, in fact, something they should do with the mediation of digital tools. On the other hand, farmers are not dispossessed of their prominent role in taking the final decision. This is the way in which they are *interested*, in line with the rhetoric of most industry observers that, according to Duncan et al. (2021, p. 1193), consider DSS “as merely supplementing farmer decisions,” rather than a substitute for their own decision-making processes.

Hence, technological improvement might foster a radical change not only in the labour conditions of farmers, but also in their identity and role, as for the value of their expertise and experiential knowledge. Indeed, this is consistent with the narratives that can be found in trade journals, as is shown by Duncan et al. (2021, p. 1188, original emphasis): a quotation that reads as “While *precision agriculture is the brain of the farmer*, a drone can be the body of the farmer” offers an example of how PA, as a set of tools, is defined in industry and trade. This is, however, also indicative of the potential substitution of farmers' knowledge with the information derived from digital tools (Wolf and Wood, 1997).

**Figure 2:**
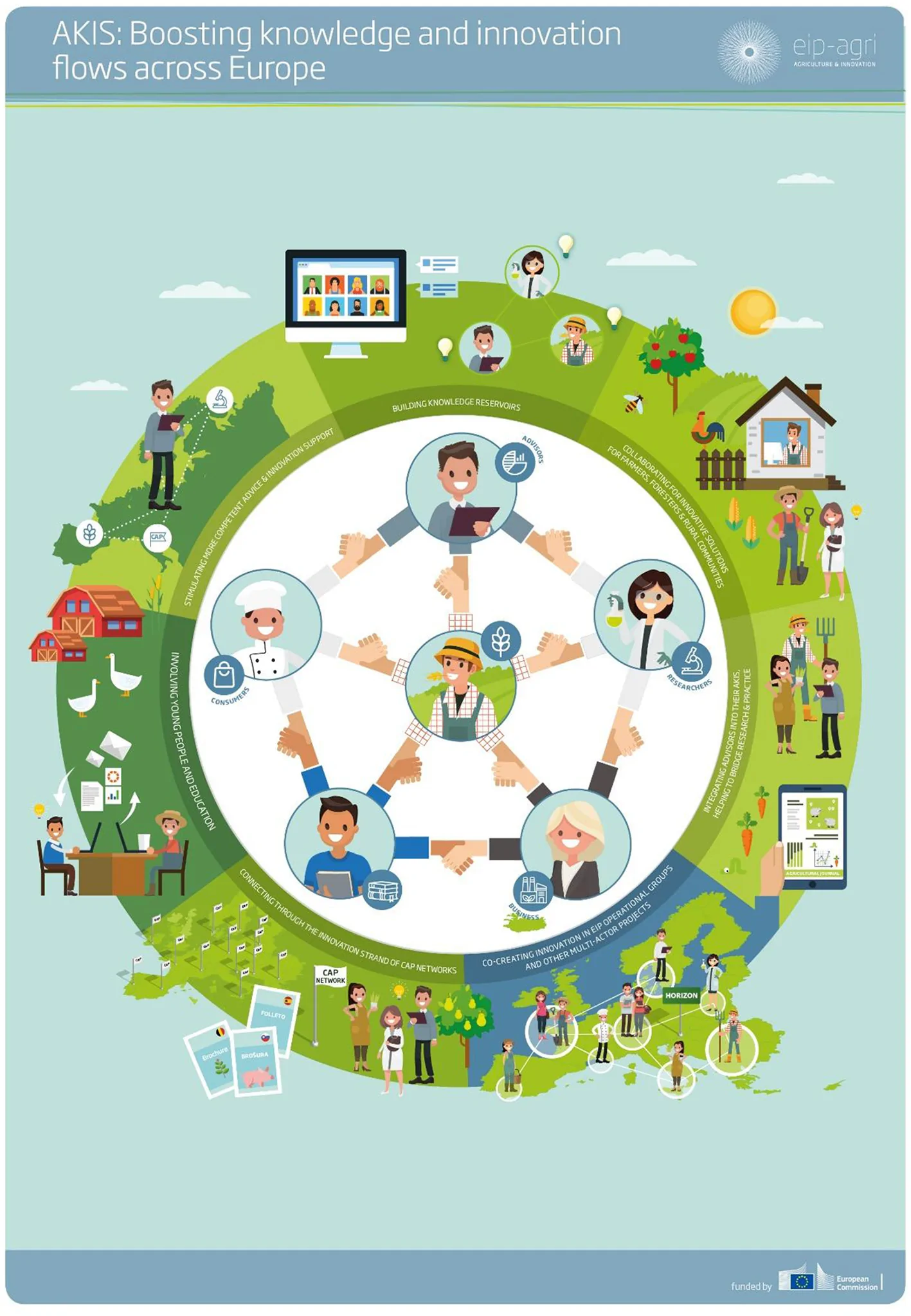
EIP-AGRI infographic “AKIS: Boosting knowledge and innovation flows across Europe.” ©EU CAP Network; available at: https://eu-cap-network.ec.europa.eu/publications/eip-agri-brochure-agricultural-knowledge-and-innovation-systems-boosting-innovation_en.

If technology should be the farmer's aid in managing the complexity of their tasks, farms and farmers need to be supported in this transition. In fact, EU policy frameworks also aim to implement knowledge sharing systems that involve farmers. This idea is fostered by the “Agricultural Knowledge and Innovation Systems” (AKIS) developed under the European Innovation Partnership for Agricultural Productivity and Sustainability (EIP-AGRI), launched in 2012 to contribute to the Europe 2020 Strategy for smart, sustainable and inclusive growth (Defour, 2021). AKIS can be identified as a formalised outcome of the associations that experts' discourse tends to offer about a vision of the farmer as an actor who is embedded in a system of relations with other heterogeneous actors from different domains and specialties, as illustrated in Figure 2.

As the infographic in Figure 2 shows, the farmer is placed at the centre of a system of relations that involves different kinds of actors, activities, and sectors. In the farmer's close network there are researchers, advisors, businesses, students and consumers. In the larger circle, a diverse array of heterogeneous networks and professional communities—including rural communities—is depicted, dealing with science, education, innovation, and denoting a complex web of interrelations that involve farmers and connect them to what Anthony Giddens termed “expert systems” (Giddens, 1990). In this way, such a “network of networks” has the capacity to redefine agricultural practices.^7^

It is evident that, as with numerous other specialised activities, farming was associated with expert systems long before the emergence of digital agriculture. Nevertheless, it is anticipated that farmers will become integrated into these systems in a variety of novel ways, largely due to the influence of digital technologies and the broader concept of smart farming, also in accordance with EU regulations and specifically the CAP. Indeed, this necessitates the provision of support to farmers as they navigate a route to innovation that is perceived to be both promising and intricate. In Event 2, a researcher emphasised the necessity of providing support and stewardship to farmers in order to address the complexity of the CAP. Moreover, they made reference to AKIS, associating it with the provision of assistance to farmers in the digital and ecological transitions (Defour, 2021):

Why do I emphasise support? Because *we are asking for a change so profound that without support*... [it cannot be pursued]. […] [We need] an approach that puts the farmer back at the centre and that sees *everyone around the farmer*: consultants, public institutions, society, trade associations, everyone to try not only to identify an innovation that is adequate but also a support that makes production successful (researcher, Event 2, audio transcript).

Assisting farmers in changing their views is a claimed objective of a trade association as well, as one of its representatives said: “Our role is to accompany the farmer in the agriculture of the future.” Analogously, EU experts also stress that economic sustainability needs to be coupled with environmental sustainability, and that training farmers to address the transition is crucial and needs specific endeavours:

The Farm Advisory System makes innovative knowledge and practices accessible to all farmers. “We are facing a major transformation of the agricultural sector that requires a shift towards more environmentally-friendly practices without undermining economic sustainability” explains Tassos Haniotis, Director of Strategy, Simplification and Policy Analysis in the European Commission's Directorate General for Agriculture and Rural Development. “The Farm Advisory services could take the form of experimenting with new practices, training of farmers, linking with universities or research institutes and providing to them with the data necessary to run their experiments with new techniques” (https://digital-strategy.ec.europa.eu/en/library/achieving-agricultural-sustainability-through-farm-advisory-system-and-rural-digitalisation, accessed 17/01/2026).

The above examples illustrate how farmers are positioned in different forms of public discourse, placing them at the centre of innovation networks, which involve complex expert systems that must be embedded in agricultural practices in order to oversee and transform them. Furthermore, Event 2 saw scientists taking on the role of protagonists, participating in the promotion of digital agriculture and contributing to farmers' understanding of the CAP. Importantly, in this endeavour, they were attempting to instruct farmers on how to comply with regulations and embrace the digital and ecological transition.

However, it should also be noted that the stances of those representatives of trade associations and experts (and of politicians too) towards the CAP are quite controversial. In fact, they expressed several concerns regarding the constraints that the CAP's directives impose upon farmers' business. At the same time, the representatives wanted to indicate how to exploit the relevant funding opportunities. In this respect, their discourse obviously differs from that of European institutions, which consistently advocate for strict compliance with the regulation, albeit concurrently promoting the sustainable intensification of agriculture.

Specifically, the discourses of these speakers are ambivalent. On the one hand, they criticise EU policy lines, stressing that these have to take care of farmers' interests and needs: “we must once again make sure that [EU's policymakers] write policies that make sense and that can improve the environment, improve the quality of life, but understanding that it is a path which is taken together with farmers, not against farmers” (Event 2, trade association representative, audio transcript). On the other hand, they purport an instrumental view of the CAP as it can be beneficial to farmers in terms of financial resources (e.g. subsidies). For example, another researcher, detailing the financial structure of the CAP, emphasised that “we need to understand how to get the money from this new CAP” (researcher and agronomist, Event 2, audio transcript).

Hence, such representatives and experts deem important to exploit the CAP, while criticising it, and in this case such exploitation demands the preparedness of the farmers, thus proposing another role for them. To be “good farmers,” they need to make good use of the CAP.

## 5 Discussion

The development of digital agriculture has attracted critical attention from the social sciences. The present article contributes to this debate by shifting attention from the technical performance of digital agriculture to the discursive processes through which digitalization is legitimised, promoted and connected to changing understandings of farming competence and sustainability. The case study deals with digital agriculture as a distinct form of innovation within this sector. The empirical focus is two-fold: firstly, on a local geographical context, namely that of Italy's Campania; and secondly, on a wider policymaking context, that of the European Union, which governs agriculture in the relevant territories. This choice has been motivated by the increasing attention being paid to digital agriculture in industry debates, which leads to the question as to how the relevant discourses are performed in the communication pertaining to the sector.

Drawing on Callon's (1984) and Latour's (1987) sociology of translation, the study considered how trade association representatives and experts in agricultural technoscience act as spokespersons that must enrol and mobilise interested parties with an eye to ensuring that these will “follow them.” Furthermore, the study investigated how enrolment is engendered through discourses in web-based communication and information documents employed by European institutions to promote the implementation of digital agriculture in the EU. Moreover, the interpretation of empirical evidence from the study was informed by the analytical lens of the “good farmer” perspective (Burton, 2004; Burton et al., 2020).

Orchestrating the ideas that inform digital innovation—and, more generally, advanced technological innovation—in agriculture is a task of experts and policymakers who speak in the name of farmers and the non-human actors involved in farming practices. This is interpretable through the vocabulary of translation as far as the latter's result is “a situation in which certain entities control others”; these entities are those spokespersons who hold “the right to express and to represent the many silent actors of the social and natural worlds they have mobilised” (Callon, 1984, p. 224). Among these silent actors are farmers, whose interests are constantly considered in the discourses of the spokespersons and presented as a central concern for them.

Indeed, a mutual reinforcement of different ideas is evident through discursive associations between the demands of sustainable agriculture for the future of agrifood systems and farmers' interests. In all the discourses analysed in this study, adherence to sustainable agricultural practices is deemed essential, and the implementation of digital technologies is crucial for optimising resource utilisation, which is in turn indispensable for both environmental and economic sustainability. This “obligatory passage point” (OPP; Callon, 1984) renders digitalization, and the relevant experts and expert systems, indispensable in the route to innovation and, thereby, in preserving both productivity and sustainability. At the same time, this integration into new socio-economic networks raises questions concerning asymmetries of power and control over agricultural knowledge (see below). When data become central mediators of farming practices, some actors may acquire greater capacity to define what counts as valid knowledge and appropriate farming behaviour.

The process of translation also implies that several transformations and displacements occur in order that farmers' interests are brought in, and enrolment is achieved successfully (Callon, 1984). On the one hand, at the level of problematization, farmers are being encouraged to make a shift from conventional farming methods to high-tech, smart farming practices. This shift would result in a form of displacement, as farmers would be required to adjust their goals and interests to align with—and thus *follow*—those relating to sustainable and digital agricultural practices. On the other hand, this study also illustrates that the capability of farmers to take over their agricultural practices is not merely disregarded. At the level of enrolment, the contribution to sustainability is presented as a farmers' duty, which is linked to the responsibility of relying on digital tools to rationalise production. The role of digital technologies as advocated by the speakers participating in Event 2, or as it is described in EU's communication, is not one that substitutes for farmers' knowledge and expertise. However, this viewpoint is not without its ambiguities, as evidenced by the critical analysis of discourse surrounding PA (Duncan et al., 2021, p. 1194).

### 5.1 Digitalization and the reconfiguration of the “good farmer”

Indeed, the dominant narrative in the discourses analysed in this work is that digital tools can *enhance* farmers' capabilities, coupling their knowledge with the power of data to help them increase productivity and reduce inputs and costs, in line with what Duncan et al. (2021, p. 1193) highlighted in their analysis of discourses of innovation in PA. In this respect, as Duncan and colleagues note, most industry commentators claim that, in the future, “PA will give farmers ‘unprecedented control over their growing operations”' (2021, p. 1190) and that “farmers will need to become ‘techpreneurs' or ‘agricultural technologists”' (2021, p. 1193), thus granting them a prominent role provided that they will significantly change their professional identity—something that farmers can actually be aware of (Miles, 2019, p. 4).

In this sense, the hoped-for awareness of this enhancement among farmers can translate into an invitation to become “good farmers” (Burton, 2004) via the use of digital technologies. Farmers should produce “intelligently” and thereby they must acquire the skills needed to comply with the digital transition. This, in turn, will assist them in maintaining productivity (and in ensuring financial means, also by access to subsidies under the CAP). As the focus on productivity is not abandoned, being “productive” might still be a sign of “good farming” practices and could also be understood in reference to the use of digital tools. Therefore, digital competence becomes not only a technical ability but also a normative criterion through which farming competence is redefined.

Indeed, the contribution of this study is to show that digitalization is not merely framed as a technical option but as a moral and cultural marker of competent farming. Within these discourses, the “good farmer” is progressively associated with the capacity to engage with data, digital tools and expert networks. Thus, digitalization becomes part of the symbolic repertoire through which farming competence is evaluated. Indeed, these discourses do not simply describe digitalization; they contribute to shaping its trajectory by defining which forms of technological adoption appear desirable, legitimate and compatible with the identity of the “good farmer.”

The arrangement of ideas and concerns apparent from the discourses analysed highlights the relevant enrolment processes. In response to RQ1, and following Latour (1987, p. 111), the attempt to discursively enrol farmers is explicitly done by insisting that “*their usual way*” of doing agriculture “*is cut off* ”: because of climate change, and the resulting unpredictability and lack of efficiency of conventional modes of production, farmers can no longer pursue a kind of agriculture that disregards precision, efficiency, and an environmentally and economically sustainable use of inputs and natural resources (e.g., soil, water, and fertilisers, etc.). This latter point also addresses RQ2, as these non-human actors are “interested” in what is at stake and enrolled accordingly through discourse.

If farmers want to prevent themselves from economic failure and ensure the productivity of their land, they must embrace digital agriculture and thereby increase the resilience of farming practices. In addressing RQ3, it is evident that these forms of association among productivity, sustainability, and resilience are pivotal in delineating the pathway to achieving digital and ecological transition in agriculture. This transition is not merely an option but rather an imperative and a feasible objective for farmers, who would ultimately be considered “good” if they accept the new identities that policymakers and trade associations would attribute to them (i.e. responsible and skilled operators of a future, technologically advanced and sustainable agriculture).

However, it is important to acknowledge that this vision implies changes in the networks of association (Latour, 2005) among information, tools, and experience relevant to farming practices, making the latter less dependent on farmers' local and socially embedded knowledge. This standpoint would be in contrast to that of the farmers who assert that digital tools are not essential to them to be considered “good farmers” (Tsouvalis et al., 2000). Obviously, this is a matter of diversity in farmers' perceptions of digitalization in agriculture (Fleming et al., 2018; Clapp and Ruder, 2020; Duncan et al., 2021; Schnebelin et al., 2021), although the current work does not address this issue (see below for the limitations of the study).

The mentality of farmers is, in any case, called into question because of the need to abandon “traditional” agriculture and thus become a competent, “good” farmer who can manage data and digital tools. In response to RQ4, the analysis suggests that discourses on digitalization and sustainability do not simply describe the figure of the “good farmer” but actively contribute to its reconfiguration through narratives that define what forms of knowledge, skills and practices are considered appropriate within digital and ecological transitions. In the latter's respect, the current study illustrates how discourses on digitalization and sustainability construct the figure of the “good farmer” differently according to the context in which such discourses are produced. Although the discourses performed at both the European and local levels explicitly position digitalization as a fundamental element in shaping the future of agriculture, the relevant actors discursively construct the concept of the “good farmer” from a variety of perspectives. European institutional communication tends to portray farmers as knowledge-intensive actors embedded in innovation networks, where digital tools complement expertise and facilitate sustainable practices. In contrast, trade associations and local experts place greater emphasis on productivity and the practical usefulness of technologies for maintaining farm viability.

Nevertheless, being embedded in innovation networks and dealing with digital skills—which may be unfamiliar to farmers' cultural and knowledge backgrounds—could imply issues of power imbalance. Moreover, the ways in which both human and non-human actors are enrolled within these narratives suggest a performative dimension of sustainability discourse, where technologies are not merely tools but symbolic markers of progress. Such framing can obscure the socio-political implications of innovation, including issues of power asymmetry, data ownership, and dependency on proprietary platforms. These dynamics can also be interpreted as reflecting broader processes of platformization like those occurring in many other sectors, with similar tensions in terms of technopolitical implications (Van Dijck et al., 2018; Carolan, 2018; Sauvagerd et al., 2024).

### 5.2 The selective construction of ecological transition

The discourses under consideration in the present work suggest that “good” agricultural practices, insofar as they pertain to the adoption of “virtuous” modes of production, are those which link advanced data-driven monitoring of farming processes with the concomitant benefits for the environment. As noted above, the distinction between good and bad farming lies in the different associations among the entities involved, be they farmers, digital tools or chemicals. However, the discourses analysed in this study also privilege a specific understanding of sustainability, centred on efficiency, optimisation and the better management of inputs. While focusing on these aspects remains essential, this tends to marginalise alternative interpretations of ecological transition based on diversity, territorial adaptation, experiential knowledge and agroecological approaches (Schnebelin, 2022).

It is also important to note that the primary objective of PA is not to eliminate the use of pesticides, but rather to enhance their utilisation in a more efficient and effective manner. This may be interpreted in accordance with the critical reading of digital innovation proposed by Wolf and Wood (1997) and Oui (2025), who contend that precision technologies can function as a strategy to legitimise or circumvent chemically-based and intensive farming (see also Duncan et al., 2021). Moreover, the analysis highlights that digital agriculture is mainly promoted through narratives of potential and opportunity. The discourses examined in this study emphasise the benefits of digital technologies for future farming, while questions concerning their actual effectiveness, limitations and possible unintended consequences remain less visible. This aspect resonates with critical debates on precision agriculture and its possible “imprecision” (Visser et al., 2021).

Surely, the CAP and the link between sustainability and digitalization on which it is predicated demand increased awareness among farmers regarding the future of greener agriculture. However, the ways in which this is understood by the different actors involved in the transition can be varied, especially if the point is on how to cater to farmers' interests in promoting sustainable agriculture and thus encouraging them to implement the ecological transition. This point addresses RQ4 once more: while the discourses of the various categories of actors and spokespersons appear to converge on the primary objectives of preserving productivity and strengthening the competitiveness of the sector, as well as reducing workloads for future farmers, what varies in these discourses is the meaning attributed to sustainability. While EU discourses tend to connect sustainability with broader objectives of environmental preservation and rural development, the discourses of trade associations and local agricultural experts appear to frame sustainability first and foremost in economic terms. Consequently, and in line with the meaning of the word “inter-esse” (cf. Callon, 1984; Latour, 1987), environmental sustainability is identified as an objective that is instrumental in ensuring productivity and economic survival as the ultimate goals.

### 5.3 Final remarks and limitations of the study

The digitalization of agriculture has the potential to transform the relationship between farmers' knowledge and expertise and their farming practices. It is important to note that what could be regarded as an organisational or technoscientific improvement is more of a cultural change that might have significant implications for conventional farming. Indeed, this change should not be viewed as a mere consequence of digitalization; rather, it is something that farmers are *encouraged to make* by means of the device of *interessement* (Callon, 1984; Latour, 1987).

Furthermore, if one considers smart farming as the wider context in which current digital innovations are promoted, these innovations turn out to be different from merely increasing the automation of agricultural practices. In fact, it is a kind of innovation that differs from those of the past, even from the introduction of PA, especially because the increased data-driven management of information transcends mere operational terms. Relying on advanced technoscientific knowledge as a core component of these practices, current and future digital innovations clearly imply a different kind of farmers' involvement and even a different cognitive effort, which does not simply mean buying, for example, a new machine that is more powerful, faster, or more efficient. In this case, the performativity of farming concerns the relationship between farmers and the construction of knowledge about their own job, and their relationship with their land. Therefore, this also brings about changes to the socio-technical relationships that more generally preside over agricultural practices.

In conclusion, three main limitations of the present study should be acknowledged. First, the current work was based on a relatively limited ethnographic dataset, focusing on participant observation sessions conducted on a single farm and in two agricultural events, all of which were situated in a specific region of Southern Italy. The standpoint of a single farmer is by no means indicative of a broader farmer population—let alone the totality of farmers in the Campania region, Italy, or even Europe.

However, the Farmer's individual case provided the research with a valuable set of materials and key ideas that contributed to the development of the qualitative, open study design. Moreover, the triangulation of the material resulting from observations in that farm and those relating to the agricultural events allowed the author to highlight how the Farmer's point of view was akin in some respects to the concerns that experts and trade association members sought to articulate in public discourse (although denoting greater attention to agroecological practices). In any case, it is evident that the contexts of observation could be expanded to encompass a broader and more diverse range of circumstances, thereby providing a more comprehensive account of the relevant discourses.

Secondly, the investigation into policymaking has been limited to the domain of popularised communication (indeed, communication is the primary focus of the study), with issues pertinent to decision-making processes being neglected. The selection of documents from EU-related websites could also have been larger to provide evidence of a broader discourse on innovation, but this lay beyond the scope of this article.

Thirdly, and more crucially, the study does not address the perspective of farmers regarding digital agriculture, nor did it provide an opportunity to examine the efficacy of enrolling farmers in this initiative through discourse (i.e., the success of the process of translation). This significant issue may be addressed in subsequent research.

## Data Availability

The datasets presented in this article are not readily available because the data is of qualitative, discoursive nature and will be kept confidential. Requests to access the datasets should be directed to marco.serino@unina.it.

## Footnotes

1 I am grateful to a reviewer for suggesting the utilisation of this approach to analytically frame the present work.

2 Latour (1987, pp. 108–121) provides a different version of the “four moments of translation” (problematization, interessement, enrolment, and mobilisation of allies) as delineated by Callon (1984).

3 The in-person events in which the current author participated took place in Italy's Campania region. In order to guarantee the anonymity of the speakers (the majority of whom are known to the public), the exact dates of observation in these events are omitted. Furthermore, when referring to those speakers, the pronoun “they” is used to avoid identifying them by gender.

4 Documents have been accessed in Italian. In the following, however, quotations from these documents will be based on the corresponding (and often original) webpages in English.

5 The combinations are as follows. On https://commission.europa.eu/index_it: “agricoltura” AND (“digitalizzazione” OR “digitale”); on https://agriculture.ec.europa.eu/index_it: “digitalization” OR “digital”; (“tecnology” OR “technologies”) AND “innovation”; on https://digital-strategy.ec.europa.eu/it: “agriculture”.

6 It is worth noting that some of the experts who delivered speeches at the conference on the CAP were collaborators of trade associations, which in fact “enrolled” them in the event.

7 Interestingly, what is depicted in Figure 2 is not only a network, but perhaps also a quite good representation of an *actor-network* (Latour, 2005).
